# The wind of change in the management of autosomal dominant polycystic kidney disease in childhood

**DOI:** 10.1007/s00467-021-04974-4

**Published:** 2021-03-07

**Authors:** Charlotte Gimpel, Carsten Bergmann, Djalila Mekahli

**Affiliations:** 1 Department of Internal Medicine IV (Nephrology), Medical Center—University of Freiburg, Faculty of Medicine, University of Freiburg, Hugstetter Str. 55, 79106 Freiburg, Germany; 2 Medizinisches Versorgungszentrum des Klinikum Konstanz, Konstanz, Germany; 3 Medizinische Genetik Mainz, Limbach Genetics, Haifa-Allee 38, 55128 Mainz, Germany; 4 Department of Pediatric Nephrology, University Hospital of Leuven, Herestraat 49, 3000 Leuven, Belgium; 5 PKD Research Group, Pediatric Laboratory, Department of Development and Regeneration, GPURE, KU Leuven, Leuven, Belgium

**Keywords:** Autosomal dominant polycystic kidney disease (ADPKD), Children, Adolescent, Neonate, Hypertension, Proteinuria, Tolvaptan

## Abstract

Significant progress has been made in understanding the genetic basis of autosomal dominant polycystic kidney disease (ADPKD), quantifying disease manifestations in children, exploring very-early onset ADPKD as well as pharmacological delay of disease progression in adults. At least 20% of children with ADPKD have relevant, yet mainly asymptomatic disease manifestations such as hypertension or proteinuria (in line with findings in adults with ADPKD, where hypertension and cardiovascular damage precede decline in kidney function). We propose an algorithm for work-up and management based on current recommendations that integrates the need to screen regularly for hypertension and proteinuria in offspring of affected parents with different options regarding diagnostic testing, which need to be discussed with the family with regard to ethical and practical aspects. Indications and scope of genetic testing are discussed. Pharmacological management includes renin-angiotensin system blockade as first-line therapy for hypertension and proteinuria. The vasopressin receptor antagonist tolvaptan is licensed for delaying disease progression in adults with ADPKD who are likely to experience kidney failure. A clinical trial in children is currently ongoing; however, valid prediction models to identify children likely to suffer kidney failure are lacking. Non-pharmacological interventions in this population also deserve further study.

## Introduction

Gone are the days when autosomal dominant polycystic kidney disease (ADPKD) was known as “adult” rather than “autosomal dominant” polycystic kidney disease and research into many aspects of ADPKD has been very dynamic in recent years. However, because kidney failure does not occur until a median age of 55–75 years [1], clinical manifestations in children and young adults still tend to be underestimated in our experience, especially by family physicians and adult nephrologists. Combined with legitimate worries about testing for an asymptomatic disease in children, this can lead to unwarranted neglect of treatable disease manifestations in young people which jeopardizes their future kidney function. On the other hand, awareness and understanding of very-early onset (VEO) and severe forms of ADPKD have increased in the last years and enhanced our knowledge of the prevalence and genetic basis of severe childhood ADPKD. Simultaneously, the use of newly approved tolvaptan (and other disease-modifying drugs that are currently being evaluated in clinical trials) is restricted to adults with radiological criteria of rapid disease progression, which often means waiting until the kidney has already been irreversibly damaged by progressive cyst growth. Therefore, the pediatric early stages of ADPKD represent a novel and crucial target for disease understanding and treatment. Here we aim to review the spectrum of clinical manifestations of ADPKD in children, the possibilities and dilemmas of different diagnostic methods and management options in order to give an update on recent developments in this dynamic field.

## Clinical manifestation of ADPKD in childhood

### Classical ADPKD

The symptomatic manifestations of ADPKD in childhood remain difficult to grasp. Even though the prevalence of abdominal, flank, or back pain is reported between 16 and 30% [2–4], this occurs with a similar prevalence in the general pediatric population [5], and unfortunately no direct comparative studies exist. The same is true for nocturnal enuresis and urinary frequency which may be symptoms of low urinary concentrating ability [6], as well as for urinary tract infections [2–4, 7, 8]. A history of hematuria, which can be considered a more specific symptom, is reported in about 10% of pediatric case series [2, 3, 7–10]. This is consistent with investigations in adults, where 10% report a first episode of gross hematuria before the age of 16 [11].

However, asymptomatic disease manifestations are prevalent. A recent meta-analysis by Marlais et al. revealed that 20% of children with ADPKD had hypertension in 14 studies including over 900 patients (95% confidence interval: 15–27%) [12]. The incidence of hypertension increased with age in meta-regression. This fits with the increasing prevalence of hypertension reported throughout adult life in patients with ADPKD and the fact that hypertension and hypertensive vascular damage often precedes kidney damage in ADPKD [13]. Still, results are sometimes questioned because many studies of children with ADPKD are performed in tertiary centers and may have a referral bias towards more severe cases. However, the meta-analysis reports a similar rate of hypertension when removing the three studies with the highest likelihood of selection bias. Also, a direct comparative study of children diagnosed with ADPKD due to symptoms or screening, did not identify relevant differences in disease severity [3] and adult studies which included patients in their twenties show similar proportions of hypertension [14]. In addition, a 24-h blood pressure study in children with ADPKD revealed a high proportion of isolated nocturnal hypertension [15]; thus, clinic blood pressure measurements alone may even underestimate the true incidence of hypertension. Hypertensive children with ADPKD probably experience faster prospective decline of kidney function and kidney growth than their normotensive peers [16].

Hypertension in children with ADPKD is accompanied by measurable cardiovascular damage [17–19] and even those with blood pressure at the higher end of normal (75^th^ to 95^th^ percentile) already have increased left ventricular mass index compared to normotensive peers [17]. This is compatible with the finding that adults with ADPKD also suffer considerable cardiovascular damage prior to decline of kidney function which probably relates to activation of the renin-angiotensin system by ischemic cyst compression and loss of *PKD1/2*-dependent production of nitric oxide by vascular smooth muscle cells [13, 20, 21].

With regard to proteinuria, this was found in 20% (95% confidence interval: 9–40%) of 508 children in 8 studies [12]. As in adults [22], proteinuria is usually very mild or mild. The prevalence of hypertension and proteinuria appear not to correlate with each other both in the meta-analysis and within individual studies [12, 23, 24].

Reduced kidney function is not a main feature of childhood ADPKD; however, the mean risk of reduced GFR in meta-analysis is 8%, but varies widely between different pediatric case series (95% CI: 2–26%, range 2–39%) [2, 12]. Glomerular hyperfiltration, which may herald loss of GFR, can be observed in about 20% of children with ADPKD [4, 25, 26], but there is no uniform definition of this phenomenon. In a prospective pediatric study, glomerular hyperfiltration was associated with faster increase in kidney volume, as well as faster decline of GFR during follow-up [4]. This is probably the main reason why cross-sectional pediatric studies usually show no correlation between kidney volume and GFR [23], despite the fact that this is well established in adults [27].

### Extrarenal disease manifestations and distinguishing phenocopies

Hepatic cysts are only a minor concern in children with ADPKD, as single cysts are reported in only 0–4% with no cases of significant liver disease [2–4, 7–9, 23]. The same holds true for ductal plate malformation (i.e. congenital hepatic fibrosis and hyperplastic biliary ducts) which has only been described in a few anecdotal cases of ADPKD, but which is a constant feature in ARPKD and frequently observed in many other syndromic ciliopathies [28]. The incidence of cerebral aneurysms is also negligible in children, with only rare case reports. A high incidence of mitral valve prolapse (12%) in an older study [29] could not be confirmed subsequently [8]. The incidence of inguinal hernias appears quite high with around 10% in only two studies reporting on this outcome [2, 9], and was significantly higher in the group of children presenting with ADPKD_VEO_ [9].

Mutations in a large number of other genes can also mimic ADPKD and need to be taken into consideration for differential diagnosis, especially for children with negative family history (for details see [30]). For example, mutations in *HNF1B (*also known as *TCF2)* can mimic ADPKD and ADPKD_VEO,_ as *HNF1B* is involved in regulating *PKD2* expression as a transcription factor [31, 32]. However, neither hypomagnesaemia nor elevated liver enzymes are features of ADPKD, which may be a helpful diagnostic clue when considering *HNF1B-*mutation as a differential diagnosis [33]. Patients with Bardet-Biedl syndrome may also present with ADPKD-like kidney cysts, which may be caused by the involvement of BBS1 and 3 in ciliary trafficking of polycystin 1 (the *PKD1* gene product) [34]. Again, extrarenal signs such as polydactyly, obesity, or retinal disease may give helpful clues here.

### VEO, prenatal, and severe ADPKD

It has been recognized for some time that a subgroup of children have more severe ADPKD than the classical course and may even suffer kidney failure (i.e. stage 5 chronic kidney disease (CKD)) at an early age [2]. These children tend to present earlier in life with symptomatic disease that can even resemble the much more severe autosomal recessive polycystic kidney disease (ARPKD) with oligohydramnios, pulmonary hypoplasia, and consequently severe respiratory distress after birth [7, 35, 36].

Children with ADPKD_VEO_ have a higher risk of early loss of kidney function. A well-matched case-control study found that 50% of ADPKD_VEO_-children and 30% of non-VEO children progressed to eGFR < 90 ml/min/1.73 m^2^ during a long follow-up period and that this occurred at a median age of 22 vs. 25 years (*p* < 0.05) [26]. All cases of early kidney failure occurred in the VEO group. Glomerular hyperfiltration was not more common, but occurred slightly earlier in the VEO group [26]. This group also had more hypertension and very enlarged kidneys [26].

This case-control study defined VEO as diagnosis before the age of 18 months, with most patients being diagnosed in the 1990s. However, especially as pre- and postnatal ultrasound has become much more sensitive, the sole detection of hyperechogenic normal-sized kidneys in an at-risk fetus should probably not be equated to VEO disease anymore. Even though it is likely to be due to the transmission of ADPKD, it does not necessarily implicate severe disease (own data, paper in preparation). Even prenatally enlarged kidneys are often no longer enlarged in childhood [7]. The Mayo Clinic group suggests to use the term ADPKD_VEO_ when the diagnosis is made in utero with enlarged hyperechoic kidneys and/or oligohydramnios or before the age of 18 months with enlarged kidneys and one of the following: hypertension, persistent proteinuria or reduced GFR [37]. However, so far, evidence that isolated prenatally enlarged kidneys are really a risk factor for faster disease progression is lacking and it remains unclear whether some children without early onset still show rapid progression.

Genetic research has been helpful in understanding some of the large clinical variability of ADPKD. Already 25 years ago, children with the *PKD1/TSC2* contiguous gene deletion syndrome, were discovered to present with early severe ADPKD together with tuberous sclerosis complex [38]. However, this is rare as the large deletion usually occurs de novo. While homozygous *PKD1* mutations appear to be incompatible with life [39], biallelic hypomorphic *PKD1* mutations can cause severe ARPKD-like forms [40, 41], and the combination of a classical and an ultra-low penetrant *PKD1* mutation can also cause severe, early ADPKD [42]. Patients with early ADPKD are more likely to have such biallelic transmission than those with classical ADPKD [36, 41]. Digenic disease with variants in *PKD1* and the newly identified ADPKD gene *GANAB* has also recently been identified in a child with early ADPKD [43]. Severe ADPKD may also be caused by combined transmission of mutations in *PKD1 or 2* and other cystic kidney disease genes such as *HNF1B* or *PKHD1* [42], but it is unclear to what proportion of ADPKD_VEO_ children this applies.

## Understanding and stratifying disease variability

Within the group of patients with classical ADPKD, there is also considerable variability in age of onset and progression of kidney disease. Trying to identify patients at higher risk of severe disease is not only of academic interest. As some adults with ADPKD never reach kidney failure (stage 5 CKD) within a normal life span, patients who would benefit from treatments to delay disease progression need to be identified in order to save others from the unnecessary effort, risk, and cost of such treatments.

Genetic studies have been able to identify more rapid kidney growth and loss of kidney function both in adults and children with *PKD1* as opposed to *PKD2* mutations [8, 44], which are causative in approximately 80 and 15% of adult cases respectively. Consequently, truncating *PKD1* mutations were shown to cause more rapid progression than non-truncating ones [45]. Within the last 5 years, various other genes could be identified in the small percentage of patients without *PKD1* or *2* mutations: mutations in *GANAB* cause generally mild cases of ADPKD and polycystic liver disease [46], patients with *DNAJB11* variants have non-enlarged or atrophic kidneys with interstitial fibrosis [47] and mutations in *ALG9* also cause polycystic kidney and/or liver disease [48], but the further characterization of disease progression in the latter two is still lacking.

In terms of clinical markers, extensive radiological studies in adults have identified total kidney volume adjusted to height (htTKV) as good a good predictor of disease progression, which is easy to apply with the Mayo imaging classification [49], but only applicable from 16 years of age and in patients without unusual kidney shapes.

Consequently, both genotype (truncating vs. non-truncating *PKD1*, *PKD1 vs. PKD2*) and total kidney volume have been integrated into scoring systems to predict the individual likelihood of reaching kidney failure (e.g., PROPKD Score) together with clinical risk factors such as gender and hypertension or urological events before the age of 35 years [50].

Additionally, several biomarkers have been found to correlate with disease severity and progression in adults, such as plasma copeptin (a surrogate for endogenous vasopressin levels) [51], urinary MCP-1 (a chemotactic factor for circulating monocytes and a pro-inflammatory activator of macrophages) [52–54]. Urinary EGF (a marker of functional tubular cell mass and of progression in both adults and children with CKD [55]) may also be useful in ADPKD [56], but all these biomarkers have not been studied in early ADPKD.

The lack of early prognostic markers to inform long-term decision making in adults remains a dilemma for both physicians and patients [57]. In children, such knowledge is even more limited and much longer-term studies are needed to achieve the same goal.

## Establishing the diagnosis

### Asymptomatic at-risk children

**Fig. 1:**
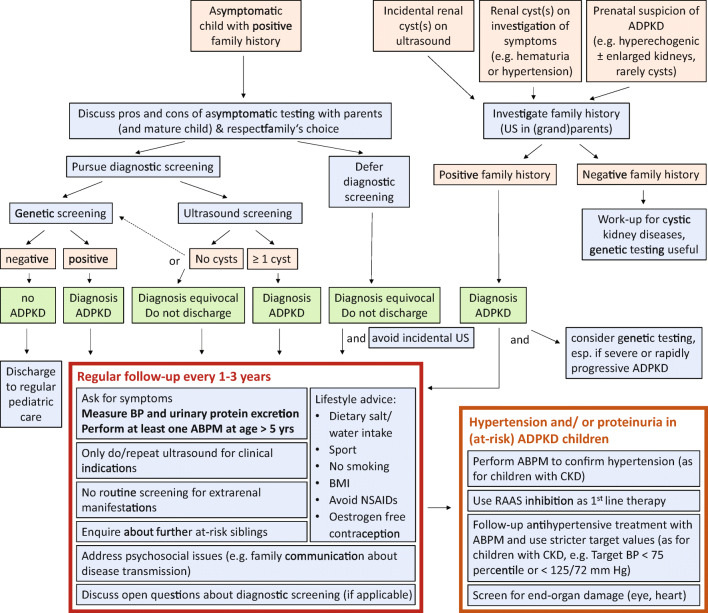
Flow chart summarizing management of children with ADPKD. Most points taken from an international consensus statement [58]. *symptoms: e.g., symptoms of urinary tract infections, urinary concentrating deficit and hypertension, abdominal pain, hematuria. ABPM: 24h ambulatory blood pressure measurement, ADPKD: autosomal dominant polycystic kidney disease, BP: blood pressure, CKD: chronic kidney disease, RAAS: renin angiotensin aldosertone system, US: ultrasound

For minors without symptoms of ADPKD who are known to be at-risk because of an affected parent, the first important management step is an in-depth discussion about the approach to diagnostic testing (see Fig. 1). While genetic testing of asymptomatic minors for untreatable adult-onset diseases is generally considered unethical [59], it is deemed permissible by both the American and European Societies of Human Genetics if treatment is available during childhood [60, 61]. Unfortunately, ADPKD does not fit neatly into such categories because children frequently have signs such as hypertension and proteinuria even without subjective symptoms, and the treatments of hypertension and proteinuria are effective, low-risk, and have proven long-term benefit, but are not strictly speaking curative. Also, it is possible to screen for hypertension and proteinuria without uncovering transmission status to children who have neither signs nor symptoms of ADPKD. However, some parents or young people may still feel a considerable psychological burden of uncertainty about transmission status and/or the need for repetitive examinations despite a 50% chance of being unaffected. Because childhood ADPKD is incurable but not untreatable, diagnostic testing can be considered, but should involve thorough prior counselling of both parents and mature children, who should be aware of all three possible management pathways illustrated in Fig. 1. Parents and the minor should understand the legal consequences that apply in their country regarding future securement of insurance policies and jobs, if a diagnosis is made in an asymptomatic person. In Germany and Belgium for example, but also in many other countries, results of pre-symptomatic genetic testing may be legally withheld from insurance companies [59], while there is no such legislation for radiological findings. The US also has a federal law which permits withholding results of presymptomatic genetic tests from health insurance companies and employers, but not from life or long-term care insurance [62], with additional heterogeneous regulations in individual states. In other countries, there may be no statutory protection, such as in the UK, where a voluntary code of conduct needs regular renewal [63]. Close interdisciplinary collaboration with a clinical geneticist is therefore helpful to inform and counsel families in navigating this challenging decision.

If diagnostic testing is agreed upon, ultrasound is a non-invasive, easy to perform and specific diagnostic tool. Even though the radiological definition of ADPKD in adults and adolescents over 16 years requires ≥ 3 cysts, in at-risk children with a positive family history even 1 cyst can be considered very likely evidence of ADPKD, as simple cysts are very rare in children and follow-up is highly likely to confirm cystic disease [64, 65]. However, especially in younger children, the absence of cysts on ultrasound is insufficient to exclude ADPKD [66] as even in adults this is not possible until the age of 40 years [65]. Genetic testing may be preferred because of higher sensitivity and legal implications in some countries. However, there is currently no added benefit of genetic testing in children with an established radiological diagnosis and classical presentation and family history, but this may change in future if disease-modifying treatment and prediction scores which include genetic subtypes are established in children. Children with a confirmed or equivocal diagnosis of ADPKD should be enrolled into regular screening for disease manifestations (see Fig. 1 and “Management” section), while only children with a negative genetic test may be safely discharged into routine pediatric care.

### Incidental cysts, symptomatic, and severe disease

For children with the incidental finding of kidney cysts on ultrasound (usually on investigation of symptoms such as abdominal pain, enuresis, urinary tract infection or hematuria), the question of family history should be addressed first (see top right-hand side of Fig. 1). This may require ultrasound examination of the parents (and grandparents if the parents are under 40 years old). A similar approach should be taken if there is prenatal suspicion of ADPKD, which is more likely to manifest with enlarged hyperechogenic fetal kidneys than visible cysts. In both scenarios, a negative family history should prompt further work-up of the presenting symptoms as well as investigations for other kidney diseases.

From the discussion on severe and early ADPKD above, it is evident that in children with severe ADPKD_VEO_ genetic analysis is usually advisable [58], as knowledge of the exact genotype helps to counsel on recurrence risk within the family and may improve the prediction of the clinical course.

### Preimplantation genetic diagnosis (PGD)

Even though this is not strictly a pediatric issue, pediatricians should be aware that PGD for ADPKD is possible if genetic studies in the affected parent are completed and conclusive before conception. Several recent publications report on PGD for ADPKD with a cumulative success rate per couple of 58–65 % [67–69]. In addition to ethical issues surrounding the procedure itself, ADPKD may be deemed a questionable indication because it is a severe, but not necessarily life-threatening or even life-limiting disease and a first disease-modifying treatment is available. On the other hand, it can be deemed especially suitable, because of the higher transmission risk than in recessive diseases and the high likelihood of identifying a causative mutation in the affected parent. In view of the wide range of attitudes towards PGD among patients affected with ADPKD and their doctors [70, 71], prospective parents deserve to be informed in order to take an independent decision [72], which will also depend on their local regulatory framework and financial coverage [73].

## Management

As illustrated in the bottom half of Fig. 1, very similar monitoring and counselling guidelines apply for at-risk children with an unknown, equivocal or confirmed diagnosis of ADPKD. Good long-term care often still requires overcoming a mindset of ADPKD as an adult disease and should be viewed as repeated opportunities for screening and counselling, two pediatric key areas of expertise.

### Monitoring and counselling

Based on the relatively high incidence of hypertension and proteinuria detailed above, two recent clinical guidelines recommend to examine urine and blood pressure in all at-risk children at regular intervals [58, 74]. Because of generally slow disease progression, large intervals of one to several years are usually sufficient and should not be overly burdensome for those children who turn out not to be affected. For school-aged children, it is worth investing the extra effort of 24-h ambulatory rather than just clinic blood pressure measurements, due to the high incidence of isolated nighttime hypertension [15], and to take the time to educate about lifestyle factors which influence blood pressure and progression of ADPKD (see Fig. 1 and section on “Non-pharmacological management”).

Even in children with an established diagnosis of ADPKD, repeated ultrasound examinations and “cyst counting” are of limited value for clinical management yet may cause significant anxiety for patients and families. Although the number of cysts and kidney volume have some correlation with hypertension, ultrasound does not replace blood pressure measurements. However, ultrasound examinations are useful in the work-up of clinical events such as urinary tract infections, hematuria, or abdominal pain. There is no need to screen children or adolescents for cerebral aneurysms or hepatic involvement, due to the extremely low incidence in young people with ADPKD.

Testing for hypertension and proteinuria can be done without revealing transmission status in children who have neither signs nor symptoms of ADPKD while children with positive findings can undergo focused work-up. It also provides repeated opportunities for families to communicate with their children about the possibility of disease transmission. Many families find this challenging, e.g., because of parental feelings of guilt or helplessness and traumatic experiences with the disease in older relatives, and may benefit from external help [75, 76]. Initiating age-appropriate communication about the risk of disease early on helps to improve family communication and improves long-term coping strategies [77, 78]. If the parents have chosen not to communicate the risk of disease transmission with their offspring during childhood, they should still be encouraged to address this issue when their children reach the age of maturity, as the ability to choose presymptomatic testing is perceived by many patients as an important and valuable opportunity to take ownership of their health [79].

### Antihypertensive and antiproteinuric treatment

Antihypertensive and antiproteinuric treatment have the dual aims of lowering cardiovascular mortality and preserving kidney function, because blood pressure is one of the important modifiable risk factors. There are universal recommendations to lower blood pressure in children with hypertension and especially children with CKD [80–83] with a host of evidence, but we will focus on reviewing ADPKD-specific evidence here.

Adults with CKD already have a large increase in cardiovascular mortality, however, patients with ADPKD are at an even higher risk of cardiovascular disease than matched patients with other causes of CKD [84, 85]. This may be due to intrarenal activation of the renin-angiotensin system by local cystic destruction and a high incidence of hypertension [13, 20]. Placebo-controlled trials are not available for patients with ADPKD, but the decrease over time of the very high incidence of left ventricular hypertrophy in this group may be attributable to the more intensive treatment approaches to hypertension [86]. In the HALT-PKD A study, more intensive lowering of blood pressure (but not single vs. dual RAS blockade) resulted in a greater decline of left ventricular mass index in adults with early ADPKD [87]. This is in line with other studies which confirm a benefit of intensive blood pressure control on cardiovascular events, especially in high risk groups [88].

Generally, proteinuric patients appear to be at greater risk of complications of hypertension and accordingly benefit more from intensive blood pressure lowering [89], although due to the combined antihypertensive and antiproteinuric effect of RAS blockade this effect is difficult to separate in many clinical trials. In the HALT-PKD B trial an ACE inhibitor alone was not different to dual RAS blockade in preventing a composite outcome of death and loss of kidney function in later stages of ADPKD, with similar blood pressure levels achieved in both groups [90].

Intensive blood pressure lowering appears to have greater benefits on cardiovascular mortality than on protection of kidney function in adults [88], and this is also true for ADPKD, where intensive antihypertensive treatment reduced growth of kidney volume but not loss of GFR [87, 91, 92]. In children, strict blood pressure control could further slow the decline of GFR in a large cohort with mixed-origin CKD [93]. The only randomized controlled trial in children with ADPKD was much smaller and stratified into three groups of children with normal (< 75th percentile), high-normal (75th–95th percentile) and high blood pressure (> 95th percentile). Children starting from a high-normal blood pressure experienced a significant decrease in GFR and increase in serum creatinine and left ventricular mass index over 5 years, which did not occur in the group treated with ACE inhibitor [16]. Children with established hypertension all received ACE inhibition and did not experience a significant benefit from intensified versus conventional blood pressure control, but a high drop-out rate and difficulty to obtain target-blood pressure make analysis of this group difficult.

In terms of drug choice, RAS inhibition with ACE inhibitor or ARB blocker is generally preferred for adults with CKD due to the additional renoprotective effects in proteinuric patients [89]. For patients with ADPKD and hypertensive children, these drugs also have a much greater body of evidence, but direct evidence of their superiority over other antihypertensives is sparse [92]. However, diuretics should probably be avoided as they are associated with a larger loss of GFR in a small retrospective study [94]. Calcium channel blockers increase cyst growth in an animal model of ADPKD [95], but studies in humans have not shown a large effect [96–98]. In adults with ADPKD, there is no convincing benefit of double RAS control versus treatment with ACE inhibition or ARB blockade alone [87].

### Pharmacological treatment to delay disease progression

This has been a very active area of research in the last decade. Many potential drugs have been identified from in vitro, animal and genetic studies, partly with extensive bioinformatic analysis [99], but we will focus here on those with pediatric data or which have been licensed in adults.

From a pediatric perspective, the fact that loss of GFR is a very gradual, continuous process, has both upsides and downsides: on the one hand, there is a theoretical benefit of starting to delay disease progression as early as possible in order to maximize the achieved delay of kidney failure [100]. Mathematical models of ADPKD progression support this idea; however, they necessarily extrapolate medium-term data to much longer time intervals [101]. On the other hand, starting pharmacological treatment early in life requires drugs with very good safety profiles. Additionally, there are several principal dilemmas for interventional pediatric ADPKD studies: firstly, GFR is not a good marker of progression in early disease (see ‘Complications of ADPKD’ above); however, even though kidney volume correlates well with kidney function and progression in adults [49], some drugs have had discrepant effects on the two and therefore kidney volume is not sufficient as a sole outcome measure [102]. Secondly, even a small reduction in the rate of disease progression would have a significant long-term effect but would require very large or very long-term studies, which are both difficult. Thirdly, in “real life” treatment of adolescents is often hampered with serious compliance problems and is more difficult in patients who feel subjectively well.

Nonetheless, in children with ADPKD who already received ACE inhibition, a prospective randomized controlled trial of pravastatin could show a significant reduction of height-adjusted kidney growth over 3 years [103]. There was also a non-significant reduction in left ventricular mass index, and kidney function was unchanged, as could be expected in a group with a mean creatinine clearance of 135 ml/min per 1.73 m^2^ over this time span. Despite this promising effect, and a plausible mode of action via the anti-proliferative, anti-inflammatory and anti-oxidant effects of statins (HMG-CoA reductase inhibitors), which improve endothelial function and increase kidney blood flow, no larger confirmative studies have been initiated. This is probably due to lack of financial incentives and because the results are less relevant to the much larger adult ADPKD population, where statins are frequently used for their well-documented positive effect on the rate of death and cardiovascular events in CKD [104]. With regard to kidney function in adults with ADPKD, two small randomized trials of statins show conflicting results [105, 106] and a recent post hoc analysis of large adult treatment trials did not show positive effects of ongoing (non-standardized) statin treatments [107]. In terms of safety of statins in children, there were no safety concerns in the 3-year ADPKD study with around 50 children who received pravastatin, but longer-term safety data are only available from a variety of small studies in children with hyperlipidemia (e.g., [108]). At least recent post hoc analyses are reassuring about the safety of combined use of tolvaptan and statins in adults [109].

The vasopressin 2 receptor antagonist tolvaptan is thought to reduce cyst growth by blocking the effect of arginine vasopressin on collecting duct cells, which otherwise transmits proliferative stimuli via cyclic AMP and promotes secretion of fluids into the cyst lumen via the cystic fibrosis transmembrane conductance regulator (CFTR) chloride channel. Clinically, tolvaptan causes the picture of renal diabetes insipidus with polyuria due to loss of free water. In a large randomized trial in adults (TEMPO 3:4), tolvaptan has been shown to reduce the rate of GFR loss as well as growth of total kidney volume in adults with ADPKD [90, 110] and has consequently become the first licensed drug for delaying disease progression, but only for adults with rapidly progressive disease. Difficulties of treatment include aquaretic side effects such as large polyuria, thirst and nocturia, which were experienced by over 65% of patients and led 7% to discontinue the drug [111]. Idiosyncratic liver damage is another issue, affecting about 1.2% of patients in TEMPO 3:4 and 3.7% in the extension study TEMPO 4:4. It appears to be a specific but reversible side effect in ADPKD which warrants regular control of liver function enzymes [112]. Another important unresolved question, which is particularly relevant to patients with early ADPKD, is whether the effect of tolvaptan really is sustained in the long-term. While it was continuous over the 3-year period of TEMPO 3:4, the follow-up study up to 5 years was not totally conclusive on this [113–115].

From TEMPO 3:4, there is reason to believe that young patients benefit from tolvaptan too. Two post-hoc analyses in the subgroups of patients with CKD Stage 1 and in 18–24 year olds showed significantly slower growth of kidney volume with treatment compared to placebo [116, 117]. However, decrease of GFR decline was not significant in both. Patients with CKD 1 and 2 had less hypernatremia than those with CKD 3, and were not more likely to suffer liver damage [116]. Patients with CKD stage 1 had the highest rates of polyuria or study withdrawal due to adverse events [111, 116]. Results of the ongoing first pediatric study of tolvaptan in adolescents are therefore eagerly awaited and can be expected soon [118] (ClinicalTrials.gov identifier: NCT02964273). However, as an initial study, the primary endpoints have been defined as urine osmolarity changes and change in kidney volume is only a secondary endpoint. Further study will also be required to define which children are likely to experience rapid progression and would therefore benefit from treatment. The difficulty of this task is illustrated by that fact that for adults with ADPKD different definitions/ recommendations across the US, Europe, and Japan result in widely varying groups of eligible patients [119].

A case report has documented the use of tolvaptan in a neonate with severe, recessive-like ADPKD, edema, hyponatremia, and compression of the inferior vena cava [120] with successful resolution of edema and hyponatremia and lack of further kidney growth without significant side-effects over 17 months.

Seeing as tolvaptan therapy is unlikely to be tolerated lifelong and treatments with fewer side-effects would be preferable, it is promising that there are currently 5–10 other ongoing interventional clinical studies for drugs as diverse as metformin, tyrosine kinase inhibitors, proglitazone, venglustat (oral glucosyl-ceramide synthase inhibitor), lixivaptan (non-peptide vasopressin 2 receptor antagonist) as well as prescribed water intake and caloric restriction diet [121]. Previous experience with drugs which appeared promising, such as mTOR inhibitors and somatostatin analogues, but were not found to preserve kidney function in humans (despite effects on kidney growth and liver cysts respectively [51, 52]) cautions against too much enthusiasm, but there is hope that further treatments will become available in due course.

### Non-pharmacological management

Higher salt intake is prospectively associated with worse kidney outcomes in the general population [122], as well as with higher kidney volume and growth in ADPKD [123, 124]. A recent study could also demonstrate a negative effect of salt intake on prospective GFR decline in adults with ADPKD with a wide range of eGFR [125], while in patients with advanced ADPKD it has been shown to increase the risk for a composite renal endpoint including death [124]. While in the general CKD population the negative effects of sodium intake are probably mediated via increased blood pressure and activation of the renin-angiotensin system, in ADPKD sodium-induced increase of vasopressin is a likely additional mechanism leading to cyst growth [125]. Unfortunately, there is a lack of prospective interventional trials [126], but counselling patients with ADPKD to adhere to recommendations on salt intake appears wise, as especially younger patients often have much higher salt intake than recommended for the general population [125].

Similarly, deliberate high water intake can suppress endogenous vasopressin (and its surrogate marker copeptin) and can be assumed to be beneficial because the efficacy of tolvaptan therapy correlates with copeptin levels [51]. However, a small interventional trial suggests the opposite clinical effect [127], so results of an ongoing larger randomized trial are eagerly awaited [128]. Others have shown that a low osmolar diet (low sodium, low protein and adjusted water intake) can decrease copeptin in adults with ADPKD [129], but protein intake itself did not correlate with GFR decline in ADPKD [125]. Also, protein restriction has potential side effects in growing children and could not be shown to slow GFR decline in a pediatric group of mixed-cause CKD [130]; therefore, low osmolar diet and protein restriction should not be recommended for children with ADPKD.

In addition to counseling families on lifestyle factors that affect cardiovascular risk, such as exercise and avoiding obesity and smoking, it is useful to point out patient organizations, which exist both internationally and in several countries. The interactive self-management tool “ADPKD Patient route map” [131, 132] is based on a multidisciplinary position statement and available in several languages.

## Perspectives for pediatric ADPKD research

The host of adult trials investigating interventions to delay progression of ADPKD has brought exciting perspectives for pediatric patients too. However, prior to the use of disease modifying drugs in children with ADPKD, it is necessary to establish reliable prediction models for estimating lifetime risk of kidney failure applicable to this population. The long delay between childhood and average age of kidney failure makes this a difficult task. While some factors can probably be inferred from adults, such as genotype (truncating vs. non-truncating *PKD1*, *PKD1* vs*. PKD2*), early onset of hypertension or urological events [50], these have not been validated in children, and probably need to be refined further. For example, radiological features need adjustment as enlargement of total kidney volume is uncommon in pediatric ADPKD, MRI is more difficult in children who need sedation, but ultrasound is feasible more often because of smaller kidneys. Several studies have already identified a link between larger kidneys and higher blood pressure in children with ADPKD [15, 17, 23, 133]. However, a predictive effect has been examined only for blood pressure on increase of kidney volume [23, 133], but not vice versa. Also, further knowledge on the course of glomerular hyperfiltration and genetic modifiers may be especially relevant to predicting disease progression for those who have not yet suffered clinical events or loss of kidney function. It is hoped that a new international registry for children with ADPKD (ADPedKD, www.adpedkd.org) will pave the way to larger-scale longitudinal follow-up studies and improve our understanding about the most informative predictive factors for clinical outcomes in children [134].

In parallel, the ongoing trial on the effect of tolvaptan in children is an important step forward to studying disease modifying agents for pediatric ADPKD. However, less industry-driven research into the effects of statins in children and non-pharmacological interventions such as low-salt and high-water intake is also needed. If further agents are effective in adults, their pediatric use will have to be considered.

## Conclusion

Progress on understanding disease manifestations of ADPKD in childhood means that children with an affected parent should no longer be ignored in the medical management and be regularly screened for hypertension and proteinuria. Pediatric nephrologists can help to spread this wind of change by raising awareness among the wider medical community and affected families, who are often pre-occupied with more severe disease manifestations in older relatives. Considering the high prevalence of cardiovascular morbidity in adults with ADPKD, which often precedes loss of kidney function, opportunities for early control of hypertension and establishing healthy lifestyle habits should not be missed. Diagnostic testing of at-risk children in families with classical ADPKD should not be rushed into, but repeated counselling about issues involved can enable a process of shared decision-making between the child, parents and physicians.

Pharmacological treatments to delay progression of ADPKD provide hopeful prospects for patients. However, before tolvaptan or other drugs can be used in children with ADPKD, valid prediction models are urgently needed to select appropriate patient populations and the long-term safety and sustained effect need to be verified.
